# Formation of amyloid fibrils in vitro by human γD-crystallin and its isolated domains

**Published:** 2008-01-16

**Authors:** Katerina Papanikolopoulou, Ishara Mills-Henry, Shannon L. Thol, Yongting Wang, Abby A.R. Gross, Daniel A. Kirschner, Sean M. Decatur, Jonathan King

**Affiliations:** 1 Department of Materials Science and Technology, University of Crete, Heraklion, Crete, Greece; 2 Massachusetts Institute of Technology, Department of Biology, Cambridge, MA; 3 Boston College, Biology Department, Boston, MA; 4 Mount Holyoke College, Department of Chemistry, South Hadley, MA

## Abstract

**Purpose:**

Amyloid fibrils are associated with a variety of human protein misfolding and protein deposition diseases. Previous studies have shown that bovine crystallins form amyloid fibers under denaturing conditions and amyloid fibers accumulate in the lens of mice carrying mutations in crystallin genes. Within differentiating lens fiber cells, crystallins may be exposed to low pH lysosome compartments. We have investigated whether human γD-crystallin forms amyloid fibrils in vitro, when exposed to low pH partially denaturing conditions.

**Methods:**

Human γD-crystallin expressed and purified from *E. coli*, is stable and soluble at 37 °C, pH7, and refolds from the fully denatured state back to the native state under these conditions. Purified Human γD-crystallin as well as its isolated NH2- and COOH-terminal domains were incubated at acid pH and subsequently examined by transmission electron microscopy, absorption spectroscopy in the presence of Congo red, FTIR, and low-angle X-ray scattering.

**Results:**

Incubation of the intact protein at 37 °C in 50 mM acetate buffer pH 3 at 50 mg/ml for 2 days, led to formation of a viscous, gel-like solution. Examination of negatively stained samples by transmission electron microscopy revealed linear, non-branching fibrils of variable lengths, with widths ranging from 15 to 35 nm. Incubation with the dye Congo red generated the spectral red shift associated with dye binding to amyloid. Low-angle X-ray scattering from samples showed clear meridional reflection at 4.7 Å and a more diffuse reflection on the equator between 10 and 11 Å which is the typical “cross-β” X-ray fiber diffraction pattern for amyloid fibers. FTIR was used to follow the evolution of the secondary structure of γD-crystallin with time during incubation of the protein at pH 3. The native protein displayed a major band at 1640 cm-1 that converted during incubation at 37 °C to a band at 1616 cm-1. An additional band at 1689 cm-1 also appeared with time. The presence of bands in the regions about 1620 cm-1 and about 1680 cm-1 has been attributed to the formation of intermolecular β-sheet structure that characterizes the fibrillar amyloid motif. The isolated NH2-terminal 1–82 and COOH-terminal 86–174 domains of HγD-crystallin also formed amyloid fibrils after incubation under the same conditions, but to a lesser extent than the full length.

**Conclusions:**

HγD-crystallin, as well as its isolated NH2-terminal 1–82 and COOH-terminal 86–174 domains of HγD-crystallin formed amyloid fibrils upon incubation at acid pH. Investigations of early stages in cataract formation within the lens will be required to assess whether amyloid fibrils play a role in the initiation of cataract in vivo.

## Introduction

The development of strategies for preventing or retarding growth of cataracts, as alternatives to surgical removal of mature cataracts, is hindered by our limited understanding of the mechanisms of lens opacification [1,2].

The transparency of the lens is largely determined by the properties of crystallins, the family of ocular lens proteins that are essential for maintaining the proper refractive index gradient needed for the focus of light onto the retina [3]. The crystallins within the central nucleus of the lens have to remain in their native conformation for a lifetime since these lens fiber cells are post-mitotic and lose their biosynthetic machinery during the differentiation process. The crystallins are present at high concentrations (200–450 mg/ml) and arranged in a closely packed environment [4-6].

The βγ-crystallins are structural proteins of the lens while the α-crystallins possess molecular chaperone properties and are able to interact with unfolded proteins in response to stress and to prevent their aggregation [7-12]. Human γD-crystallin (HγD-Crys) is the third most abundantly expressed γ-crystallin in the lens and contains 173 amino acids [13,14]. X-ray crystallographic studies at 1.25 Å resolution showed that the monomeric protein adopts the characteristic fold of the βγ-crystallin superfamily [15]. The protein consists of two highly homologous domains, each composed of two β-sheet Greek key motifs. The NH2-terminal and COOH-terminal domains (N-td and C-td) are joined together by a six-amino acid linker sequence and interact through side chain contacts at the domain interface [16]. HγD-Crys possesses differential domain stability with the C-td being more stable than the N-td [17]. Equilibrium unfolding/refolding experiments at near-physiologic conditions (pH 7 at 37 °C) demonstrated that the protein refolds through the sequential structuring of its domains, with the C-td refolding first [17]. Domain interface residues subsequently nucleate the refolding of the N-td. Equilibrium unfolding/refolding experiments also revealed the presence of a partially folded intermediate probably containing the C-td in its native conformation and the N-td in its random state [18].

During cataract development, insoluble aggregates of all three classes of crystallins accumulate in the lens and lead to light scattering and loss of lens clarity [19]. Protein precipitation and aggregation might occur by changes of the properties of crystallins driven by point mutations or age-related post-translational modifications. There are several types of hereditary cataracts that have been linked to mutations of human crystallin genes [20-26]. For example in HγD-Crys single amino acid substitutions are associated with juvenile-onset cataracts, which may be mediated through spontaneous crystallization or disulfide cross-linked aggregation of the mutants [27,28]. Lens opacity in these cases is, therefore, associated with reduction in the solubility of the native state and the formation of solid state complexes [29,30].

For mature-onset cataract, the mechanisms of aggregation in the aging lens are unlikely to be the same as for the rare inherited juvenile-onset cataracts. The crystallins of aged and cataractous lenses present a variety of covalent changes including deamidation [31,32], oxidation of methionine and cysteine residues, backbone cleavage [32], and glycation [33]. The consequence of these modifications may be conformational changes that alter the crystallin stability, leading to their denaturation and aggregation. Mature-onset cataract could be considered as a conformational disorder [22,34] where protein deposition may be driven by the formation of a partially folded intermediate [35,36].

At least 20 human protein misfolding and protein deposition diseases are characterized by the accumulation in different tissues of amyloid fibrils. The different pathologies are believed to arise from a common mechanism [36,37]. In each case, there is a change in the conformation of a normally folded protein, which leads to the formation of a partially folded intermediate from which amyloid fibrils can be formed by auto assembly [38-40]. The destabilization of the protein can be produced by mutations or by the introduction of partially denaturing conditions [41]. Amyloid is defined by three features, namely its tinctorial affinity for the dye Congo red, its unbranched fibrillar appearance upon analysis by electron microscopy and its characteristic “cross-β” X-ray diffraction pattern [36,37,42]. The ability of polypeptide chains to form amyloid structures is not limited to the disease-associated proteins. Proteins that are not known to be amyloidogenic in vivo have been shown to undergo self-assembly into fibrils in vitro [43-49].

Experiments performed under denaturing conditions with wild-type bovine crystallins demonstrated that all three classes of proteins are able to form amyloid fibers [50]. In mice Sandilands and coworkers [52] have shown that mutant truncated forms of the γ-crystallins form inclusions containing filamentous material in the lens that bind Congo red. Furthermore the purified truncated protein formed amyloid fibrils in vitro [51]. In vitro filament-like formation has also been identified upon interaction between α-crystallin and βL-crystallin.

Previous studies of the unfolding/refolding pathway of HγD-Crys in guanidine hydrochloride at 37 °C and pH 7 revealed an in vitro aggregation pathway that proceeded via the formation of partially folded species [53]. The aggregates were ordered with filamentous appearance, as seen by atomic force microscopy and could bind bisANS. In the present studies we demonstrate that at low pH in vitro γD-crystallin and its isolated domains polymerize into fibrillar aggregates. Using a combination of Congo red binding, Fourier transform infrared spectroscopy, X-ray fiber diffraction and electron microscopy approaches we present direct evidence that the fibrils are amyloid in character.

## Methods

### Protein expression and purification

The HγD-Crys, N-td and C-td coding sequences were cloned into the pQE.1 plasmid (Qiagen, Valencia, CA) that added an NH_2_-terminal 6-His Tag to the proteins. Bacterial expression and purification of the recombinant proteins has been described in a previous paper [17]. Briefly, protein expression was induced at 37 °C by addition of IPTG. After cell lysis by sonication and removal of the insoluble material by centrifugation, the supernatant was loaded onto a Ni-NTA column. The purified proteins were stored at 4 °C in 10 mM ammonium acetate pH 7 buffer.

### Fibril preparation

To generate fibril samples, the proteins were incubated at 37 °C in 50 mM acetate buffer pH 3 at 5 mg/ml for 2 days. In these conditions the soluble crystallin proteins aggregated and formed a gel that, as we show in the Results, consists of amyloid fibrils. Fibril formation can be induced at pH 3 using a protein concentration as low as 50 μg/ml.

### Electron microscopy

Samples of the proteins incubated at 37 °C in 50 mM acetate buffer pH 3 at 5 mg/ml for 2 days, were diluted to 1 mg/ml and deposited onto glow-discharged carbon-coated, formvar-filmed copper grids. They were subsequently negatively stained with 1% uranyl acetate and viewed in a JEOL 1200 transmission electron microscope. The dimensions of the fibrils were obtained directly from the micrographs.

### Congo red staining

Samples were tested for Congo red binding by the spectroscopic band-shift assay [54]. Fibrils were formed as for electron microscopy and diluted into 500 mM sodium phosphate buffer pH 7 at a 0.5 mg/ml final concentration. Twenty-five μl of a 100 μM freshly prepared stock solution of Congo red were added to 475 μl of protein solution and spectra were recorded from 400 to 600 nm. Absorption spectra of protein samples in the absence of the dye were also collected at the same wavelengths, to subtract the scattering contribution of the aggregates from the spectrum of the dye in their presence.

### FTIR measurements

HgD-Crys was dialyzed against deuterated 50 mM sodium citrate buffer pH 3 at a concentration of 20 mg/ml. The sample was placed between two CaF2 windows separated by a 100 mm spacer and placed within a water-jacketed cell. Sample temperature was held at 37 °C via a circulating water bath. FTIR spectra were measured on a Bruker Vector-22 FTIR spectrometer Each spectrum consisted of an average of 512 scans recorded with 4 cm-1 resolution. Spectra were measured every 30 min for up to 20 h. A spectrum of the blank buffer (also recorded at 37 °C) and of water vapor were subtracted from the protein absorbance spectrum.

### X-ray fiber diffraction

Each pre-incubated protein fiber sample was prepared for X-ray diffraction, using two different methods. For the first preparation, each protein solution at a concentration of 5 mg/ml was aspirated into a 0.7 mm diameter siliconized thin wall glass capillary tube. The capillary tubes were sealed at the narrow end by flame. The wide end of the capillary was sealed with wax through which a pinhole was punched using a hot needle. The peptide solution in the capillary tube (which stood vertically) was then allowed gradually to dry under ambient temperature and humidity until the formation of a small, uniform birefringent disk. For the second preparation, 5–8 μl of protein fiber sample were deposited between the ends of two glass rods and air-dried. These samples were loaded into glass capillaries to be examined by X-ray diffraction. Measurements at room temperature from the samples were conducted using the Oxford diffraction Xcalibur PX Ultra system (Oxford Diffraction Ltd., Concord,MA) located in the laboratory of Dr. A. Andrew Bohm (Department of Biochemistry, Tufts University, Boston, MA) [55]. The CuKα X-ray beam was generated using an Enhance Ultra, which is a sealed tube-based system incorporating confocal multilayer optics. The X-ray beam was monochromated and the Kβ component was removed by means of the double bounce within the confocal optics. The X-ray beam was focused to 0.3 mm x 0.3 mm (full-width at half-maximum width at detector position). A two-dimensional Onyx CCD detector (Oxford Diffraction Inc., Concord, MA) was placed 85 mm from the sample position, covering the scattering range of Bragg spacing 1.8 Å-54 Å. The sample-to-detector distance was calibrated by a spherical ylid crystal (C_10_H_10_SO_4_) or a cubic alum crystal according to the information given by manufacturer. The active range of the detector was 165 mm, and the two dimensional image (1024×1024 pixels; in 2×2 binning) was collected using the software package CrysAlis (CrysAlis CCD and RED, version 171 [2004], Oxford, UK) and stored in the compressed image format IMG. Exposure time was 150–300 s. The diffraction image in the form of JPG as supplied by CrysAlis RED was translated to TIFF, and then displayed by NIH image software (developed at the USA National Institutes of Health). Using the known Bragg peaks of the fundamental period of 58.38 Å from silver behenate, the pixel size of the image was calculated as 121 μm for both the specimen to film distance of 85 mm.

## Results

### Fibril formation as seen by electron microscopy

**Figure 1:**
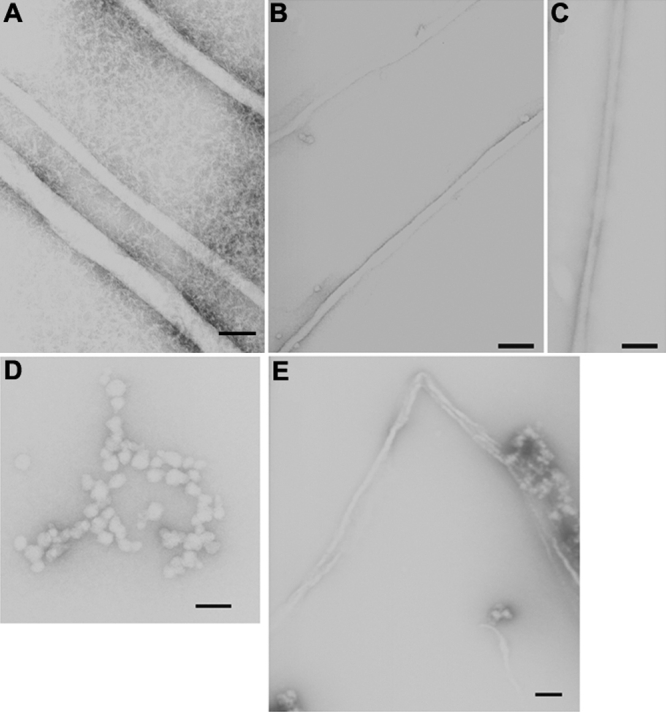
Electron micrographs of fibrils negatively stained with 1% uranyl acetate. Conditions were as follows: **A**: 5 mg/ml solution of the HγD-Crys into 50 mM acetate buffer pH 3 incubated at 37 °C for 2 days, **B**: 5 mg/ml solution of the HγD-Crys Ctd into 50 mM acetate buffer pH 3 incubated at 37 °C for 2 days, **C**: 5 mg/ml solution of the HγD-Crys Ntd into 50 mM acetate buffer pH 3 incubated at 37 °C for 2 days, **D**: 50 μg/ml solution of the HγD-Crys into 100 mM sodium citrate pH3 deposited on the grid after 2 h of incubation at 37 °C, **E**: 50 μg/ml solution of the HγD-Crys into 100 mM sodium citrate pH3 deposited on the grid after 6 h of incubation at 37 °C. The bar represents 1,000 Å.

Complete wild type HγD-Crys as well as its N-td and C-td domains, were expressed and purified from *E. coli* strains. All three preparations were predominantly monomeric when characterized by liquid chromatography. On incubating the three crystallin proteins, namely HγD-Crys, HγD-Crys N-td and HγD-Crys C-td, at pH 3 and 37 °C, we observed the formation of a viscous, gel-like solution after a few hours. This conversion was associated with the presence of unbranched, long fibrils as revealed by electron microscopy of negatively stained samples. Figure 1A-C shows electron micrographs that were recorded after incubation of 5 mg/ml solutions for 2 days, for each of the three proteins. The fibrils display typical amyloid morphology. Their diameter ranged from 15 to 35 nm, similar to the width of fibrillar structures formed from other amyloidogenic proteins [39]. These results suggest that at pH 3 the proteins fail to remain correctly folded but instead they convert to insoluble fibrils through the disruption of their normal protein conformation.

The morphological development of the fibrils was better observed at protein concentrations as low as 50 μg/ml. The process of fibril formation started with small bead-like structures that have already been described in a variety of amyloidogenic systems [56-66]. After 6–8 h of incubation these prefibrillar aggregates could still be seen but in addition, structurally well defined species with fibrillar morphology began to appear. Figure 1D,E shows representative fields from grids where a drop of a 50 μg/ml HγD-Crys solution was deposited after 2 and 6 h of incubation in pH 3 at 37 °C. In high-concentration gel-like samples these oligomeric species were not present, suggesting their transformation to fibrils.

### Congo red binding

**Figure 2:**
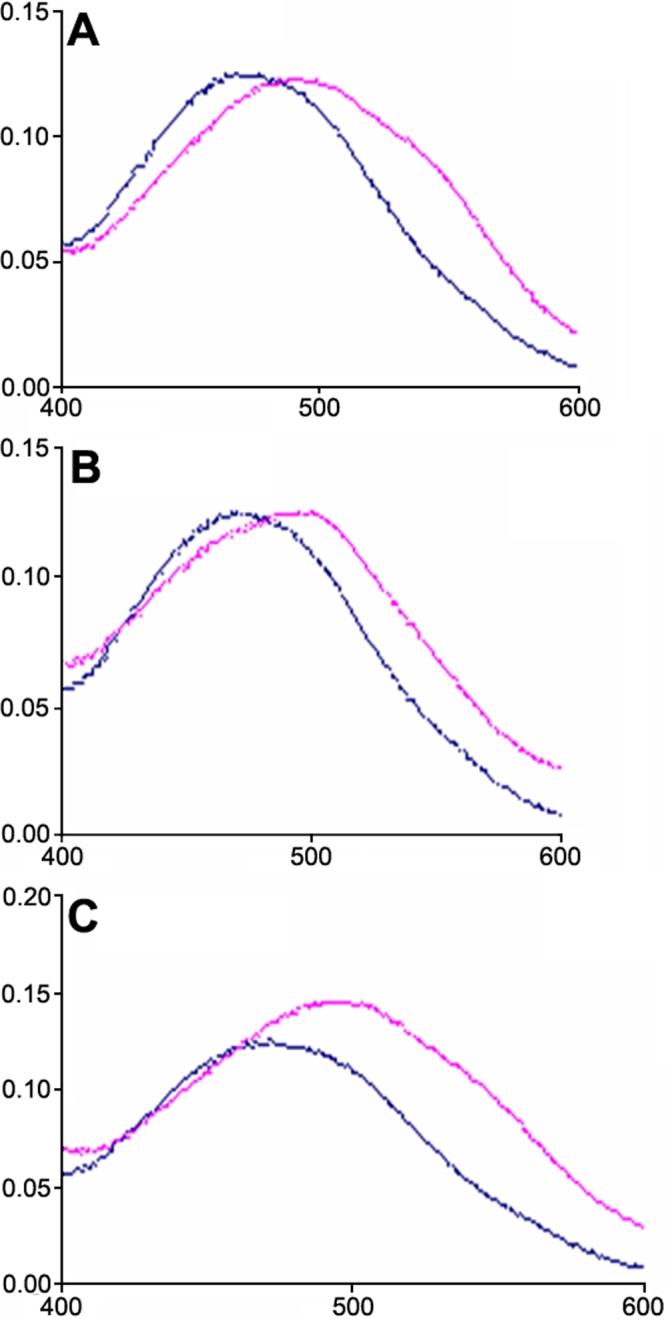
Congo red binding to crystallin fibrils. The spectra of 5 μM Congo red in the absence (blue line) and in the presence (red line) of fibrils formed by **A**: HγD-Crys, **B**: HγD-Crys Ctd, and **C**: HγD-Crys Ntd. Before adding the dye, the scattering of the peptide solutions was recorded and subtracted from the spectrum of the dye in their presence. Fibrils were formed by incubation of the proteins at 37 °C in 50 mM acetate buffer pH 3 at 5 mg/ml for 2 days and subsequently diluted in a 500 mM sodium phosphate buffer pH 7 at a 0.5 mg/ml concentration.

Congo red is a diazo dye that interacts specifically with highly ordered cross β-sheet aggregates. Congo red birefringence is considered to be one of the three hallmarks of amyloid. The binding of the dye is revealed by the spectral difference between fibril-containing solutions and dye-only solution [54]. The absorbance maximum of the Congo red incubated in buffer alone increases and shifts to red upon interaction with ordered fibrils. Figure 2 shows the dye spectra in the presence of aggregates formed from HγD-Crys, HγD-Crys N-td, and HγD-Crys C-td. For the dye-binding assay the samples were diluted into pH 7 buffer, which did not solubilize the polymerized protein. The addition of the dye to samples containing crystallin fibrils produced the characteristic red-shift expected from interaction with amyloid fibrils.

### Secondary structure of the peptides in their fibrillar state as determined by FTIR

**Figure 3:**
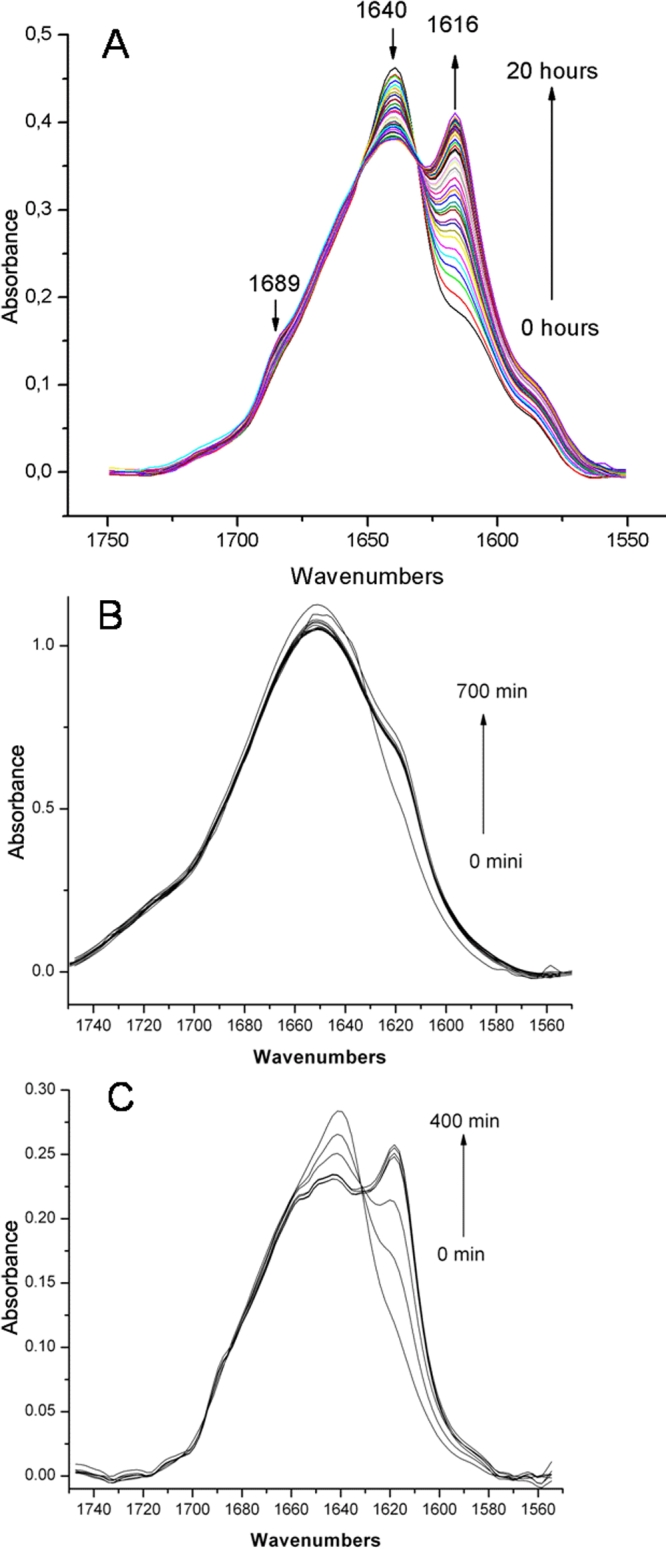
FTIR spectra as a function of incubation time from 0 to 20 h, at low pH, 37 °C. **A**: HγD-Crys, **B**: HγD-Crys Ctd, and **C**: HγD-Crys Ntd. The spectral shift associated with fibril formation is indicative of an increase in the extent of antiparallel β-sheet content.

FTIR is a key method for the study of the secondary structure of protein aggregates. Using this technique, we followed the structural transition of HγD-Crys with time during incubation of the protein in pH 3. The IR spectrum in the amide I' region of native HγD-Crys contains a single broad band, with maximum about 1638 cm-1, consistent with the spectra of other globular, β-sheet rich proteins. After incubation at pH 3, two new amide I' bands appear, at about 1616 and about 1689 cm-1. These bands are characteristic of the flat, extensive antiparallel β-sheets found in fibrous aggregates [67]. The transition from the native spectrum to the amyloid-type spectrum was monitored by collection of spectra at various time points, after shift to acid pH. The transition has a clear isosbestic point (Figure 3A).

The isolated, native N-td and C-td domain had similar IR spectra as the full length HγD-Crys. When incubated at pH 3, the IR spectra of both of these domains also developed new amide I' bands at 1616 cm-1 and 1689 cm-1, though to a smaller extent than seen in the full length HγD-Crys (Figure 3B,C).

### X-ray fiber diffraction

**Figure 4:**
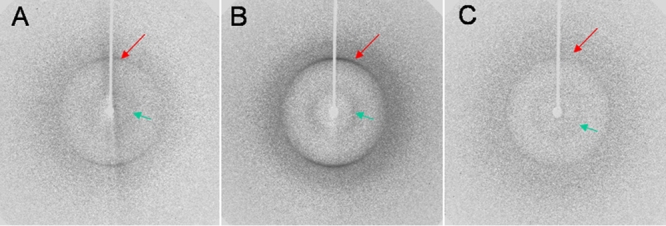
X-ray fiber diffraction from crystallin fibrils. X-ray fiber diffraction patterns recorded for the **A**: HγD-Crys, **B**: HγD-Crys Ctd, and **C**: HγD-Crys Ntd fibrils showing the characteristic features associated with the cross-β amyloid motif: an H-bonding 4.7 Å (long arrow) meridional reflection and an about 10 Å broad reflection (short arrow) on the equator. See experimental procedures for sample preparation.

Fibrils produced in acidic conditions were also examined by X-ray fiber diffraction. The recorded diffraction patterns for each crystallin protein are shown in Figure 4. All three of them are characterized by the presence of a clear meridional reflection at 4.7 Å and a more diffuse reflection on the equator between 10 and 11 Å. These two reflections are consistent with a β-structure where the β-sheets are spaced by about 10 Å and arranged parallel to the fiber axis with their β-strands lying perpendicular to that direction. The 4.7 Å reflection on the meridian arises from the separation between adjacent strands. The two dominant features constitute the “cross-β” diffraction pattern which along with evidence from electron microscopy and Congo red staining, has come to be considered as diagnostic for amyloid type structures [39,68].

## Discussion

The native states of the γ-crystallins are highly soluble and highly stable, showing little or no tendency to polymerize under physiologic conditions. The aggregated states populated during refolding or unfolding in vitro, as with many other protein deposition diseases, are formed of partially unfolded species [17,18,50]. The conformation of the crystallin polypeptide chains that aggregate during refolding at pH7 in vitro are partially unfolded molecules with the COOH-terminal domain native-like and the NH_2_-terminal domain disordered. Within the lens such partially unfolded crystallins could be generated by oxidative covalent damage or other environmental stresses. However, the conformations of the aggregated crystallin chains within a mature cataract, or the chains that are precursors to the cataract, have not been characterized.

One aggregated state that is associated with a variety of human diseases is the amyloid fibril [69]. Though mature cataracts do not resemble amyloid plaques, very little is known of the early stage of cataract formation. Meehan and coworkers [50] showed that all three classes of bovine crystallins formed amyloid fibers in vitro, under a particular set of partially denaturing conditions. In mice, a truncated form of γ-crystallin formed inclusions within lens fiber cells that exhibited amyloid character, and the purified truncated protein formed amyloid fibers in vitro upon dilution from the denatured state [51]. These observations led us to examine the propensity of human γD-crystallin to form amyloid fibers. Though low pH environments have not been reported in the lens nucleus, the differentiating epithelial and fiber cells have lysosomes, which presumably maintain the low pH denaturing environment found in other cells.

The results reported here establish that human γD-crystallin forms amyloid fibrils upon incubation at acid pH. Since the full-length protein is composed of two homologous domains, a domain swapping mechanism would provide one model for the polymerization process [70]. However, the isolated COOH-terminal domains also formed amyloid fibers in reasonable yield. This suggests that the Greek key fold itself reorganizes to form the amyloid structure.

Electron microscopy of cataractous lenses in mature onset cataract [71,83,84] do not exhibit the morphological features displayed by amyloid plaques [72]. However, cataracts are generally removed after they have grown to macroscopic dimensions. The early stages have not been identified within the lens. It remains possible that the initiating or nucleating species have a different character than the final bulk aggregate.

Many proteins have been shown to form amyloid fibrils under physiologic or non-physiologic conditions [36,43,45,46]. Thus the formation of amyloid fibrils in vitro does not establish that such reactions might be relevant within the lens. However there is some evidence for the formation of amyloid fibers in both mouse and human lens.

Goldstein and coworkers [73] have reported the presence of amyloid inclusions in cataracts from individuals with Alzheimer disease. The supranuclear cataracts colocalized with enhanced Aβ immunoreactivity and birefringent Congo red staining. In vitro, Aβ was found to promote lens protein aggregation with curvilinear protofibrillar structure. It also has been noted that early-onset cataracts and Alzheimer disease are typical comorbid disorders in adults with Down syndrome [74]. These results raised the possibility that formation of amyloid fibers by crystallins or by other proteins within the lens might stimulate non-amyloid cataract growth.

The lens grows through differentiation of lens epithelial cells into elongated fiber cells. These elongated cells initially have the full spectrum of cell organelles including lysosomes. In fact because of the need to degrade cellular organelles the outer fiber cells at some stage must have very active proteolytic apparatus. The activity of the ubiquitin degradation system has been well documented in lens cells [75]. In the case of amyloid disease due to mutations affecting transthyretin, Kelly and coworkers [41,76] have proposed that the amyloidogenic partially-unfolded intermediate is generated during degradation in the lysosome. Crystallins targeted to lysosomes due to oxidative or photo-oxidative damage might generate amyloidogenic intermediates during breakdown in the lysosomes of outer fiber cells. Since amyloid is very stable under physiologic conditions, these fibers might play a role in nucleating other aggregated states later in the life of the lens.

Recent studies indicate that the relatively disorganized prefibrillar aggregates have greater toxicity to cells than mature fibrils [69,77-81] and consequently it has been suggested that fibril formation could offer, during the early stages of the diseases, a protection against the toxic prefibrillar intermediates [59,69]. In the lens, the damaging effects of cataract, whether or not they include amyloid components, are most likely due to the direct role of large aggregates in scattering light and interfering with image formation. However, disruption of fiber cell organization has been described in cataractous lens [82-84]. Answers to some of these questions will require studying early stages in the formation of cataracts in situ, before they are large enough to interfere with lens transparency.
